# Books and Authors

**Published:** 1907-05

**Authors:** 


**Report of the Commissioner of Education** for the year ending June 30, 1904. In two volumes. Washington: Government Printing Office. 1906.

The two volumes comprising this report present an exhibit of the educational status throughout the world for the year ending June 30, 1904. It shows that in the United States the number enrolled in schools and colleges, public and private was 17,896,890, an increase over the previous year of 357,422 pupils. It will be observed that the school enrollment comprises about one-fifth of the total population of the United States. The number in attendance upon special schools, public and private, in addition to the enrollment in the schools and colleges above mentioned, is 693,101. Adding this number to the aggregate in attendance on the schools and colleges (17,896,890) gives general education in this country a grand aggregate of 18,589,991. This splendid showing has not been equalled by any other country in the world. The report contains, *inter alia,* an account of education at the Louisana Purchase Exposition, including an address on the lessons of the exposition by Dr. Howard J. Rogers, first assistant Commissioner of Education, state of New York, chief of the department of education and director of the congresses of the exposition; an interesting account of "The battle against alcohol," the latter being from the French of F. Dupré La Tour; temperance instruction in public schools, as well as many other instructive papers and reports.

All in all it is one of the most interesting and valuable of the recent reports of the department of education.

**The Technic of Operations upon the Intestines and Stomach.** By **Alfred H. Gould,** M.D., Boston, Massachusetts. Octavo volume, containing 190 original illustrations, some in colors. Philadelphia and London: W. B. Saunders Company, 1906. (Cloth, $5.00 net; half morocco, $6.00 net).

The technic elaborated in this book is the result of experimental studies on animals, mainly dogs, the work being repeated on the cadaver for anatomical corrections. These studies, which are most beautifully illustrated throughout the text, embrace three years of research work, and describe both in text and illustration, certain standard operations on the stomach and intestines with artistic and scientific exactitude. It is a book worthy the author and publishers; it supplies the operator with the information regarding technic that he may stand in need of at a critical moment; and, moreover, occupies a field heretofore practically vacant. It is a pleasure to possess such a book. It is in strong contrast to many books sent out that are of little or no value to medicine or surgery. This is a contribution to surgical literature that will endure, and no surgeon will permit himself to be long without it. We miss in the index of names that of Davis. It is well known to every informed surgeon that the Davis'—John D. S.—and William E. B.—were among the pioneers in the study of intestinal anastomosis in this country. Their contributions to the literature are too well understood and appreciated to make this omission of special significance.

---

**Transactions of the American Surgical Association.** Vol. 24. Edited by **Richard H. Harte,** M.D., Recorder of the Association. Philadelphia: William J. Dornan, Printer.

The transactions of this great surgical organisation, always interesting, are unusually so in this instance. Dr. VanderVeer's presidential address on the duality of malignant manifestations, carcinoma and sarcoma in the same individual is an interesting clinical contribution to this important subject. The cases he reports point directly toward the existence of this curious exhibition of cancerous lesions. A further attraction is Professor Trendelenburg's illustrated paper on the surgical treatment of epispadias and ectopy of the bladder. The paper by Mr. Charles A. Ballance, of London, in which he gave some experiences in intracranial surgery is still another valuable contribution to that very important topic and adds to the instructiveness of the volume. Mr. Hamilton A. Ballance, of Norwich, Eng., reported a case of traumatic hemorrhage into the left lateral lobe of the cerebellum treated by operation with recovery, which still again serves to make a group of attractive foreign papers.

The volume is full of interest, containing many other papers of value by the members, but we must content ourselves with mentioning these few, because lack of time and space forbid a general review of this excellent book.

**The Medical Directory of New York, New Jersey and Connecticut.** Published by the Medical Society of the State of New York. Volume VIII. Edited by **Wisner R. Townsend,** M.D., secretary, 1906.

In accordance with a resolution passed by the house of delegates May 19, 1906, only registered physicians in the State of New York are included in this directory. In New Jersey and Connecticut, of course, this rule does not apply. Since the union of the association and society the directory heretofore published by the Medical Society of the County of New York has been discontinued.

This issue contains the names of 15,531 physicians, 11,976 of whom reside in the state of New York, 2,276 in the state of New Jersey, and 1,279 in the state of Connecticut. Of the number given as residing in the state of New York 4,293 belong in Manhattan and the Bronx, 1,559 reside in Brooklyn, 124 in Queens, and 58 to Richmond—a total for the municipality of Greater New York of 6,034. The number, therefore, residing in the remainder of the state is 5,942. This issue follows the handsome lines of its predecessors.

---

**Studies in the Psychology of Sex—Erotic Symbolism, The Mechanism of Detumescence, The Psychic State of Pregnancy.** By **Havelock Ellis.** Vol. 5. Pages x-285. Sold only by subscription to physicians, lawyers, and scientists. F. A. Davis Company, Philadelphia. ($2.00 net).

This last volume of these studies is the most scientific, in some respects, of any of the series. Especially is this the case of the section on the mechanism of detumescence wherein the phenomena of discharge of energy in conjugation, together with its associate psychic manifestations, is delineated with sufficient accuracy to meet the purpose of the author. He justly remarks that the consideration of this phase of his topic carries investigation into the fields both of anatomy and physiology. The author has met this condition with great skill, imparting useful information with delicacy of expression and at the same time omitting no important or essential fact. By a stretch of the immagination he makes "detumescence" stand for "orgasm", which is well, because this book goes to the audience for which it was written with greater propriety of expression.

---

**A Textbook on the Practice of Gynecology.** By **W. Easterly Ashton,** M.D., LL.D., Professor of Gynecology in the Medico-Chirurgical College of Philadelphia. Third edition. Octavo of 1096 pages, with 1057 original drawings. Philadelphia and London: W. B. Saunders Company, 1906. (Cloth, $6.50 net; half morocco, $7.50 net).

The practical character of Ashton's treatise is indicated by the fact that a third edition was needed within a year after its first publication. Alterations and changes have been made to meet the advances in methods, so that the book represents the present acquirements of gynecological science. We think it unnecessary to enumerate these additions or changes but may express the opinion that, as at present constituted, it is one of the great works on gynecology. Its original illustrations constitute no unimportant part of its excellence and its author may well feel proud of his work which has cost time and a considerable money outlay.

---

**A Textbook of Obstetrics.** By **Barton Cooke Hirst,** M.D., Professor of Obstetrics in the University of Pennsylvania. Fifth edition. Octavo of 915 pages, with 753 illustrations, 39 in colors. Philadelphia and London: W. B. Saunders Company, 1906. (Cloth, $5.00 net; half morocco, $6.00 net).

Hirst has been recognised for some years as one of the leading authorities on obstetrics. His treatise, published first in November, 1898, and now in its fifth edition, has become a conceded authority and is listed as a textbook in the leading medical schools.

This edition has been carefully revised and represents the best line of teaching on this most important branch of medical science, especial attention being paid to puerperal infection and gestational toxemia. These are among the most important topics that engage the attention of obstetricians at the present day, hence deserve treatment at the hands of an experienced teacher like Hirst.

---

**A Textbook of Pathology.** By **Alfred Stengel,** M.D., Professor of Clinical Medicine in the University of Pennsylvania. Fifth revised edition. Octavo of 977 pages, with 399 text-illustrations, many in colors, and 7 full-page colored plates. Philadelphia and London: W. B. Saunders Company. 1906. (Cloth, $5.00; half morocco, $6.00, net prices).

So excellent a work as Stengel's needs little attention at the hands of a reviewer and it is merely necessary to call attention to the improvements which have been made in this, the fifth edition. A large part of the section dealing with general pathology has been reconstructed and it is now in most complete form. There is noticeable improvement in the chapters on inflammation and animal parasites. While the book as a whole has been increased in size its general character as a textbook for students has not been lost sight of and it is issued as the most perfect type of such a work which has yet appeared bearing this author's name.

---

**The Immediate Care of the Injured.** By **Albert S. S. Morrow,** M.D., Attending Surgeon to the Workhouse Hospital and to the New York City Home for the Aged and Infirm. Octavo of 340 pages, with 238 illustrations. Philadelphia and London: W. B. Saunders Company, 1906. (Cloth, $2.50 net).

Among the semi-scientific books which are designed for the home or the factory office rather than for the medical man, this occupies a prominent place. The writer's idea has been to prepare a book which would be useful to the layman and he has been eminently successful. In addition it would serve excellently as a textbook for first aid classes. To this end simplicity of language is a feature and technical terms have been used only where absolutely necessary. There is a large number of illustrations, many of them original. One of the chief charms of the book is its lucidity and its intelligence. It is not above the everyday reader and in this it is valuable.

---

**The Practitioner's Medical Dictionary.** An Illustrated Dictionary of Medicine and Allied Subjects by **George M. Gould**, A.M., M.D., Editor of "American Medicine." With 388 illustrations. Octavo; xvi-1043 pages. Philadelphia: P. Blakiston's Son & Co. (Flexible leather, gilt edges, rounded corners, $5.00; with thumb index, $6.00, net).

This edition of Gould's dictionary is an improvement over the volumes which have gone before, in that it has been brought accurately up to date. There is little on which to base honest criticism. The newest words find place in its pages and one of the features is the introduction of the terms of the Basle anatomic nomenclature. The book is excellently published; good paper, clear type, irreproachable letter press and splendid illustrations being special points. A very gratifying feature of the mechanical work is the method of binding which permits the book to lie flat open at any page.

---

**Thornton's Pocket Medical Formulary.** New (8th) edition, revised to accord with the new U. S. Pharmacopeia. Containing about 2,000 prescriptions with indications for their use. Lea Brothers & Co., Philadelphia and New York. 1907. (Leather, $1.50 net).

So popular is Thornton's Formulary that it has gone into the eighth edition and the new volume just received shows radical improvement over its predecessors. The author has arranged diseases alphabetically and under each is given the best formulas for all the conditions which might arise. One of the chief values of the book is the rearrangement necessitated by the recent changes in the pharmcopeia. The importance of these changes is inestimable; even if the formulas are not made use of. The information contained in the book regarding the changes in strength and preparation of drugs, would make it almost invaluable for ready reference.

---

**Manual of Artificial Limbs.** Copiously illustrated. Octavo, pp. 430. A. A. Marks, 701 Broadway, New York.

The house of A. A. Marks is, doubtless, the most extensive as well as the most scientific manufacturer of artificial limbs within the borders of the United States; nor is it excelled in these respects anywhere in the civilised world.

This book is properly called a Manual; it is divided into chapters devoted to the several phases of the subject; and is properly illustrated, so that those seeking information can readily find it, no matter upon what special point it is desired. It will thus be easily understood that we are not dealing with a catalogue of mechanical appliances, but a treatise upon artificial limbs. It would be difficult to find any form of amputation stump or deformity not mentioned. It is a book well worth examining.

---

**A Textbook of Pharmacology.** Including Therapeutics, Materia Medica, Pharmacy, Prescription-Writing, Toxicology, etc. By **Torald Sollmann,** M.D., Assistant Professor of Pharmacology and Materia Medica, Western Reserve University, Cleveland, Ohio. Second edition. Octavo of 1070 pages, illustrated. Philadelphia and London: W. B. Saunders Company. 1906. (Cloth, $4.00; half-morocco $5.00, net prices).

Activity in pharmacologic research since the publication of the first edition of this book has made necessary the issue of another volume which is practically a new work because a complete rewriting has been a necessity. The chapter on laboratory work has been recast entirely and now includes apparatus and technic. As an indication of the completeness of the volume there is a dose table for animals. There are many new illustrations.

---

**Saunders's Hand-Atlases.** Atlas and Epitome of Dentistry. By **Prof. Gustav Preiswerk,** of Basil. Edited, with additions, by **Geo. W. Warren,** M.D., Professor of Operative Dentistry at the Pennsylvania College of Dental Surgery. With 44 lithographic plates in colors, 152 text-illustrations, and 350 pages of text. Philadelphia and London: W. B. Saunders Company. 1906. (Cloth, $3.50 net).

Dr. Warren has done an excellent bit of work in preparing this book for the Saunders series. Although it is a work on dentistry, it is nevertheless of rather more interest than such strictly technical works would be to the physician. The illustrations numbering 103 colored figures, 44 plates and over 150 text illustrations mark its chief value. The historical sketch of dentistry is intensely interesting and altogether the book is most complete.

---

**A Manual of Pathology.** By **Guthrie McConnell,** M.D., Pathologist to the St. Louis Skin and Cancer Hospital and to St. Luke's Hospital. 12mo of 523 pages, illustrated. Philadelphia and London: W. B. Saunders Company. 1906. (Flexible leather, $2.50 net).

This manual was written and published with a distinctly stated object, that of enabling the student to rapidly acquire the salient points of the subject with which it deals and this object has been achieved in every sense. There is no small work, in the manual class, which is more carefully prepared or more excellently illustrated.

---

**Manual of Clinical Chemistry,** by A. E. Austin, M.D., Professor of Chemistry and Toxicology in the Medical Department of Tufts College, Boston. 12mo, pp. 278. Boston: D. C. Heath & Co. 1907. (Price, $1.75).

Primarily this manual was intended for second year students, but it is so excellently arranged and so clear in its presentation of the elements of the subject that even first year students may secure great benefit from its study. There are many points which stand out in the arrangement of the classification which make the book the peer of others in its class.

---

**The Elements of the Science of Nutrition.** By **Graham Lusk,** Ph.D., M.A., F.R.S. (Edin.), Professor of Physiology at the University and Bellevue Hospital Medical College, New York. Octavo of 326 pages, illustrated. Philadelphia and London: W. B. Saunders Company, 1906. (Cloth, $2.50 net).

In compact form this book presents the question of dietetics in health and disease and bases it all upon sound scientific principles. For twenty years or more laboratory methods have been applied to hospital patients in Germany with a view to explaining the inner processes in disease. It is regretable that the United States is far behind in this respect and that what we do know is based on foreign research largely. The book is one which may properly be classed as a valuable addition to the library, and its methods are well worth following up.

---

**Materia Medica for Nurses.** By **Emily M. A. Stoney,** Superintendent of the Training School for Nurses at the Carney Hospital, South Boston, Mass. 12mo of 300 pages. Third edition, thoroughly revised. Philadelphia and London: W. B. Saunders Company, 1906. (Cloth, $1.50 net).

This is an excellent book for the purpose for which it is written and its value and popularity are shown by the fact that three large editions have been found necessary in a short time. There is not a nurse practising her profession who cannot get valuable hints from its pages. It is explicit, intelligible and above all interesting. Its author has the faculty of placing the subject in a readable form. The part dealing with emergencies is absolutely invaluable to the nurse.

---

**A Manual of Normal Histology and Organography.** By **Charles Hill,** Ph.D., M.D., Assistant Professor of Histology and Embryology, Northwestern University Medical School, Chicago. 12mo volume of 463 pages, with 312 illustrations. Philadelphia and London: W. B. Saunders Company, 1906. (Flexible leather, $2.00 net).

Written for elementary students this book places the subject before them in a new light and the fundamental facts are stated in as clear and concise a manner as possible. The illustrations are helpful. A valuable section is that on the oral cavity in which the structure and function of the teeth are fully discussed. Each subject is treated in a fundamental manner, but completely, if briefly.

---

**Retinoscopy in the Determination of Refraction at One Meter Distance with the Plane Mirror.** By **James Thorington,** M.D., Author of "Ref-action and How to Refract," etc. Fifth edition. pp. 67. With 54 illustrations. Philadelphia: P. Blakiston's Son & Co. 1906.

Revised and enlarged and improved in many ways this fifth edition is so unlike the previous issues of the work that it is almost unrecognisable. There are 54 illustrations in this edition many of which are in colors. A great deal of attention has been paid to the newer instruments including the electric retinoscope. This is a work which is well worth the time spent in studying it.

---

**Saunders's Pocket Medical Formulary.** By **William M. Powell,** M.D., author of "Essentials of Diseases of Children." Containing 1831 formulas from the best known authorities. With an appendix containing Posologic Tables, Formulas and Doses for Hypodermic Medication, Poisons and their Antidotes, Diameters of the Female Pelvis and Fetal Head, Obstetric Table, etc., etc. Eighth edition, adapted to the new (1905) Pharmacopeia. Philadelphia and London: W. B. Saunders Company, 1906. In flexible morocco, with side index, wallet and flap. ($1.75 net).

The eighth edition of this handy little volume is well up to the high standard which marked the previous issues. It is brought up to date with the changes in the pharmacopeia and besides contains many new formulas.

---

**Transactions of the twenty-eighth annual meeting of the American Laryngological Association** held at Niagara Falls, N. Y., May 31, June 1 and 2, 1906. James E. Newcomb, M.D., secretary.

This book containing the papers read at the Niagara Falls meeting of the association last year, is replete with excellent material. It gives a fair exposition of the advanced status of laryngology, the contributions, for the most part, being of the higher order. It proves an addition to the literature of the subject, which everyone who practises the specialty will welcome.

---

**A Syllabus of Materia Medica.** Compiled by **Warren Coleman,** M.D., Professor of Clinical Medicine in Cornell University Medical College. Third edition. pp. 186. New York: William Wood & Co. 1906. (Price, $1.00).

This is the third edition and has been entirely revised as necessitated by the changes in the U. S. P. The book has been entirely recast in all respects and is much superior to the preceding issues. A feature is the exclusion of all the unofficial drugs. The book is small, but it is a mine of compact information.

